# Urogenital surgery in foals

**DOI:** 10.3389/fvets.2025.1520491

**Published:** 2025-06-13

**Authors:** A. Saitua, A. Sanchez de Medina, F. Bulnes, A. Buzon, R. Miraz, D. Argüelles, E. Diez de Castro

**Affiliations:** ^1^Veterinary Teaching Hospital, University of Cordoba, Córdoba, Spain; ^2^Department of Animal Medicine and Surgery, University of Cordoba, Córdoba, Spain

**Keywords:** uroperitoneum, urachus, ureter, bladder, hernia, omphalophlebitis, umbilical

## Abstract

Urogenital surgery in foals represents a nuanced and intricate aspect of equine veterinary medicine. Disorders affecting the urinary system in newborn foals can occur at varying rates, with conditions like uroperitoneum and patent urachus being prevalent. Bladder surgeries are typically conducted through laparotomy, while laparoscopic interventions are less common. Procedures to address umbilical remnants encompass surgeries for persistent urachus or omphalitis. Rarer conditions like ectopic ureters or hydroureters may necessitate sophisticated diagnostic and therapeutic measures, including advanced imaging and minimally invasive surgical techniques, despite limited available literature on them. Post-operative complications from urogenital surgeries often involve issues associated with abdominal procedures and potential bladder closure site dehiscence, along with systemic challenges like significant electrolyte imbalances or the risk of sepsis, particularly in cases where foals have not received appropriate passive immunity transfer or pre-operative medical management. This review addresses the prevalent disorders impacting the urogenital system of neonatal foals, emphasizing their surgical treatment, potential risks, and anticipated results. The complexity of neonatal urogenital conditions requires a meticulous approach to the diagnostic work-up and therapeutic plan. Surgical approaches can range from routine to complex, requiring expert knowledge of anatomy and advanced surgical training. Complications occur and the clinician must be prepared to navigate these complications to ensure patients survival.

## 1 Introduction

Newborn foals are particularly susceptible to a vast range of urogenital disorders, with conditions such as uroperitoneum, patent urachus or omphalitis frequently encountered in clinical practice (1) Foals' unique anatomical and physiological features necessitate a comprehensive understanding of the surgeon facing these complex problems. Profound knowledge of different techniques to effectively manage the varied anatomy of ureters, kidney, bladder, or umbilical remnants pathologies is required. Also, inguinal rings, vaginal process and testes pathologies related with inguinal herniation knowledge is needed and will be covered here.

A profound review of urinary disorders of foals has been recently published (2) and is beyond the scope of this work, but the main purpose of this article is to provide a deep review of the different surgical options for management of urogenital disorders in foals. Regarding urogenital surgeries, the most commonly performed in foals include procedures that involve bladder and umbilical remnants and are traditionally performed by laparotomy. However, several laparoscopic techniques have been described, for some of those procedures as they offer valuable minimally invasive options that highlight the advancements in veterinary surgical approaches (3). In addition to these more commonly addressed issues, rarer anomalies such as ectopic ureters and hydroureters present unique challenges, often requiring sophisticated diagnostic and therapeutic measures, including advanced imaging technologies and minimally invasive techniques.

Post-operative care is as critical as the surgical procedures themselves, with potential complications often arising from the inherent risks associated with abdominal surgery, including bladder closure site dehiscence and systemic issues like electrolyte imbalances or sepsis, particularly in foals lacking adequate pre-operative medical management (2).

The complexity of these surgical interventions, which frequently address acute and life-threatening conditions should be known perfectly. Each phase of the surgical process, from correcting urologic anomalies to managing postoperative complications, demands precision, diligence, and adaptability, reflecting the critical nature of veterinary care in these young equine patients.

The objective of this review includes a thorough description of the main urogenital disorders in foals focusing on the different surgical techniques reported and comparing complications and prognosis among them.

## 2 Umbilical abnormalities: patent urachus and omphalitis

### 2.1 Patent urachus

Urachus is a tubular structure that connects the fetal bladder to the allantois, facilitating drainage of fetal urine into the allantoic cavity, with closure occurring at birth. It comprises the umbilical vein leading to the liver, the umbilical arteries flanking the bladder toward the aorta, and the urachus linking the bladder to the amniotic sac in utero, along with associated soft tissues. After birth, the internal umbilical remnants gradually regress; the umbilical vein transforms into the falciform ligament of the liver, the umbilical arteries evolve into the round ligaments of the bladder, and the urachus becomes the median ligament of the bladder (4).

A patent urachus is either congenital or acquired. A congenital patent urachus closes on its own within a few days in the absence of infection or other co-morbidities. An acquired patent urachus will have a delayed presentation and is associated with abnormalities of the umbilicus, such as septic omphalitis, omphalophlebitis, urachal abscess, or systemic compromise, resulting in increased recumbency and abnormalities of urination. Hospitalized and compromised foals may also develop patent urachus due to prolonged recumbency and urination abnormalities. In a study involving 82 foals, it was found that 18 (22%) had a congenital origin, while 64 (78%) were acquired (5).

**Figure 1:**
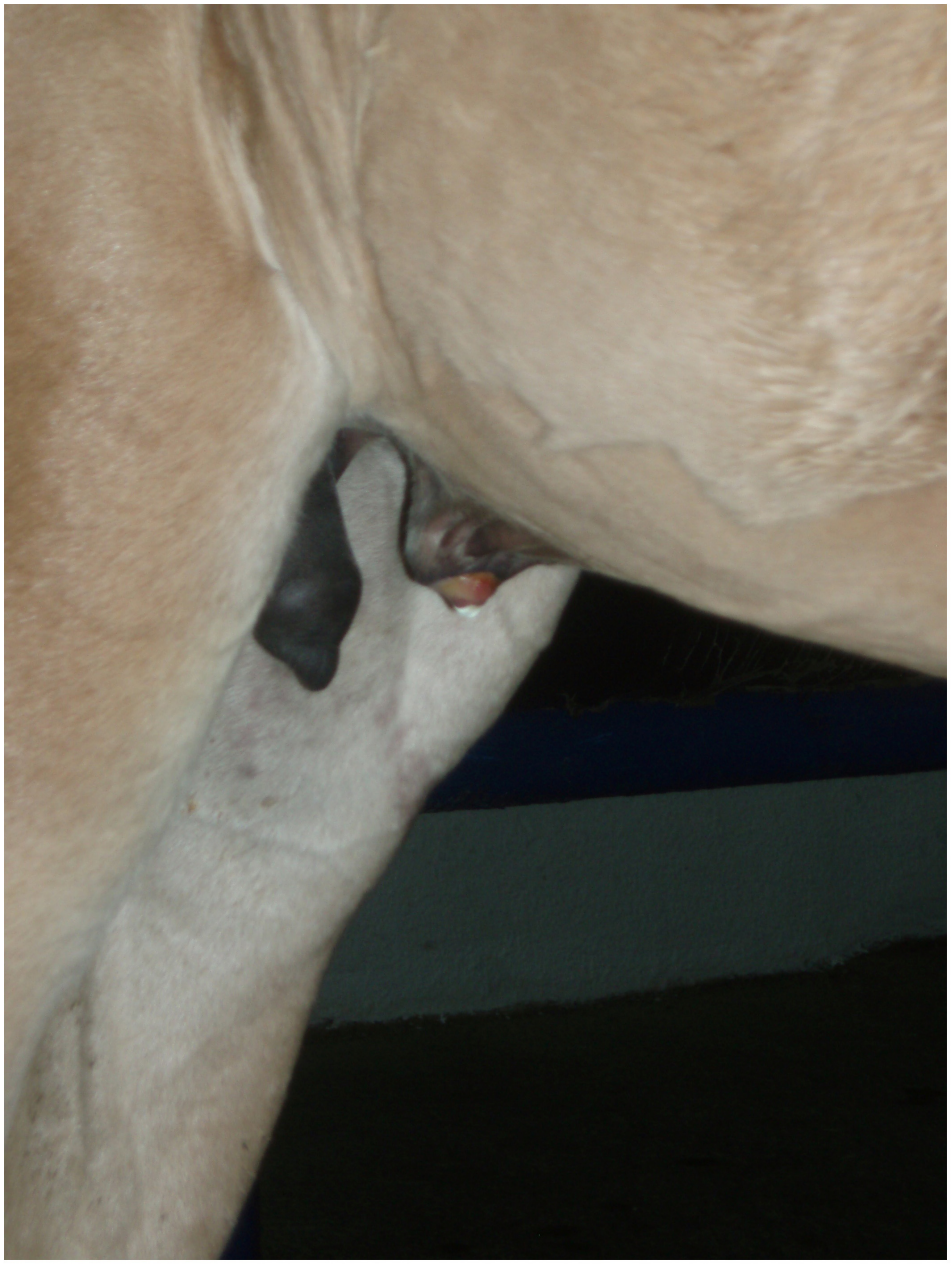
Moist umbilicus draining urine due to a patent urachus.

Diagnosis of a patent urachus can be conducted through visual inspection. The umbilicus often appears persistently moist, with urine either dripping or streaming from the urachus during urination (Figure 1). In some cases, urine leakage may not be apparent immediately after birth but can become evident within several hours or days and conditions like omphalitis or omphalophlebitis may increase the risk of a patent urachus. In these situations, a systematic, serial ultrasound is the most reliable method for achieving a definitive diagnosis. Franklin and Ferrel have proposed a protocol for evaluating these structures (6).

**Figure 2:**
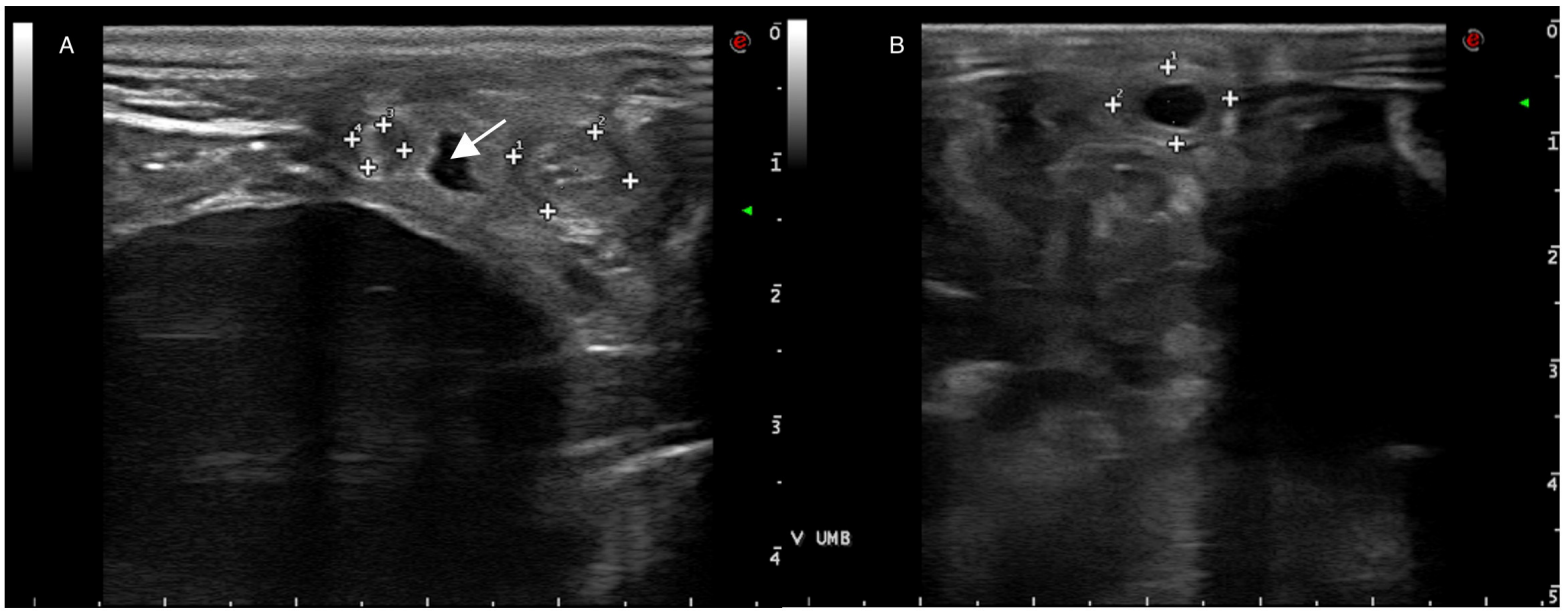
**(A)** Ultrasonography of umbilical arteries (surrounded by marks) and urachus (**arrow**) adjacent to the bladder. **(B)** Ultrasonography of the umbilical vein (surrounded by marks).

Ultrasonography of the umbilical remnants is commonly conducted in neonatal foals undergoing veterinary assessments (Figure 2). Umbilical structures (urachus, vein and arteries) can be imaged at the external remnant as it courses out of the body wall. Umbilical vein is located along the midline, close to the abdominal wall, and courses cranially from the site of the external umbilicus to the liver. It is a thin-walled structure and may contain anechoic fluid within its lumen. Paired umbilical arteries are found ventral and lateral to the urachus and bladder, coursing caudally to their origin from the iliac arteries. Urachus extends from the apex of the bladder outward to the umbilical stump, in healthy foals its lumen is typically collapsed and difficult to appreciate. However, the presence of anechoic fluid within its lumen is indicative of a patent urachus (7–9).

Dipping of the umbilicus in topical antiseptics like chlorhexidine (0.5% solution) two to three times daily is often performed in cases of congenital patent urachus to prevent secondary infection. Chemical cauterization, frequently done with 7% iodine of silver nitrate, is considered unnecessary with the risk of tissue necrosis and infection or even urachal rupture and uroperitoneum (10). Uncomplicated congenital urachus should close in a few days. Therefore, surgical treatment should be considered if urachus remains patent after 5–7 days.

For cases in which the patent urachus is acquired, systemic antimicrobial and anti-inflammatory therapy (flunixin or ketoprofen) are necessary, as well as anti-septic dipping. If possible, a sample should be taken for culture and antibiogram from the external remnant or during omphalectomy to determine the most suitable antibiotic and this treatment should persist for a minimum of 5–7 days. Frequent monitoring via visual inspection for changes in moistness or leakage during urination, serial ultrasonography to evaluate changes in size and character of the umbilical structures, and changes in clinical signs (i.e., fever, lameness or joint effusion) is essential. Worsening of these parameters may warrant surgical management to reduce the risk of complications due to urachal abnormalities such as urachal abscessation, rupture and/or uroperitoneum; as well as systemic deterioration including sepsis (2).

### 2.2 Omphalitis

Omphalitis is infection of one of more of the umbilical remnants (umbilical arteries, vein, urachus) and it has been described to be a prevalent source of morbidity in equine neonates (5, 9) within the initial weeks of life. Prevalence of omphalitis reported is quite variable depending on the source, from 0.7% in a group of healthy Thoroughbred foals in UK to 13% of foals with septicemia (11). Early identification of omphalitis is crucial as it can create significant problems for the neonate (12). Complications during pregnancy (placentitis), parturition (dystocia), or early life of the foal such as neonatal encephalopathy, failure of passive transfer, or poor hygiene of the umbilical remnant may predispose the foal to omphalitis (9, 12).

**Figure 3:**
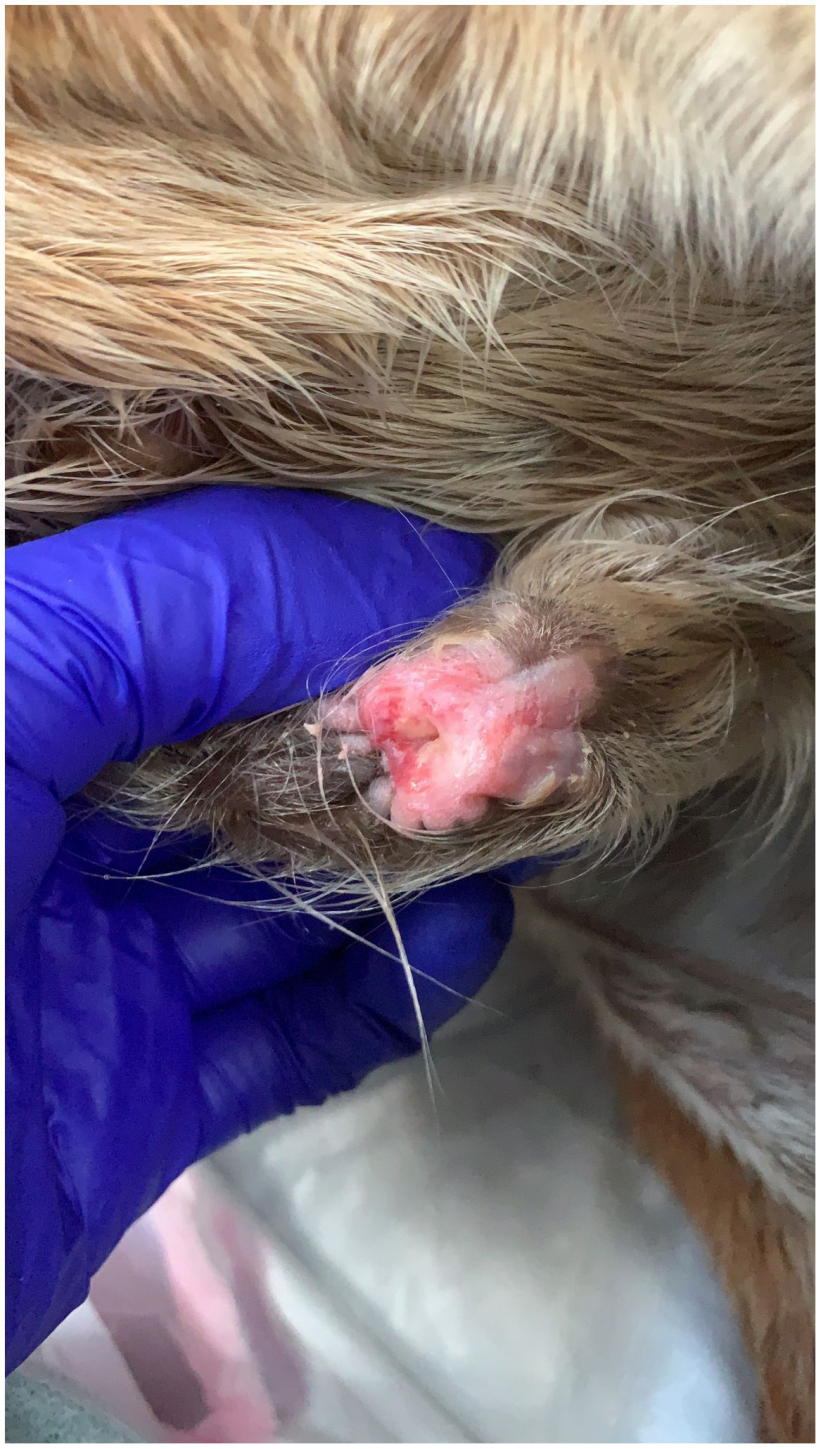
Infection of the external umbilical remnant.

Clinical signs commonly include swelling and/or discharge form the external umbilical remnant (Figure 3). The foal may develop a fever as well as an elevated white blood cell count and hyperfibrinogenemia. Omphalitis most commonly affects the urachus, but infection of one or both the umbilical arteries and the vein can occur. Infection of these structures can progress forming abscessation of the infected structure or the infection can spread locally or systemically. Abscessation of umbilical remnants may cause peritonitis and/or abdominal adhesions. Infection of the umbilical vein may ascend, spreading locally to the liver and causing liver abscessation causing greater morbidity and complicating treatment (7, 9). The infection can also cause hematogenous spread of bacteria causing septicemia and allowing further spread of bacteria to the foals' bones, joints, and/or physes. Other co-morbidities that can occur with omphalitis include pneumonia and/or diarrhea (11, 13). Due to the severity of these secondary complications, prompt diagnosis and treatment of omphalitis is essential.

Ultrasonographic evaluation of the umbilical structures is a critical step in the diagnosis of umbilical remnant infection and have an invaluable utility in selecting the appropriate surgical treatment, as it has been described to have a strong correlation with surgical findings [12, 14, 117]. Umbilical structures bigger than reference values for foal's age, together with heterogenous echogenicity or gas echoes and filling of their lumen with material of variable echogenicity and/or thickening of the wall suggest that an infection of the region probably exists (7). Although it is not always possible to determine the surgical plan based on the ultrasonographic findings, it has been described to be useful for surgery planning, especially when liver abscesses are detected (7, 15).

Treatment alternatives comprise medical management utilizing systemic broad-spectrum antimicrobials and non-steroidal anti-inflammatory therapy, either solely or in conjunction with surgical resection. Conducting culture and sensitivity testing is essential for selecting the appropriate antibiotic therapy (16), although broad-spectrum antibiotics are usually started meanwhile results of culture are available. Bacteria most frequently identified in infections associated with umbilical remnants include *E. coli, Streptococcus* spp., *Salmonella* spp., *Staphylococcus* spp., *Klebsiella* spp., *Actinobacillus* spp., and *Clostridium perfringens* (17).

An older study examined the effectiveness of umbilical abnormalities' medical treatment alone in comparison to a combination of medical treatment and surgical intervention. It emphasizes that, although the use of antibiotics and anti-inflammatories in medical treatment yields a positive outcome for mild infections, more foals survive (66%) when surgical excision is paired with medical treatment, as opposed to only 42.9% survival with medical treatment alone (18). Although the low sample size and combination of different umbilical pathologies (i.e., patent urachus and omphalitis) make that this conclusion should be taken with caution, to the authors' knowledge there is no recent publication that provides better data on the topic.

**Figure 4:**
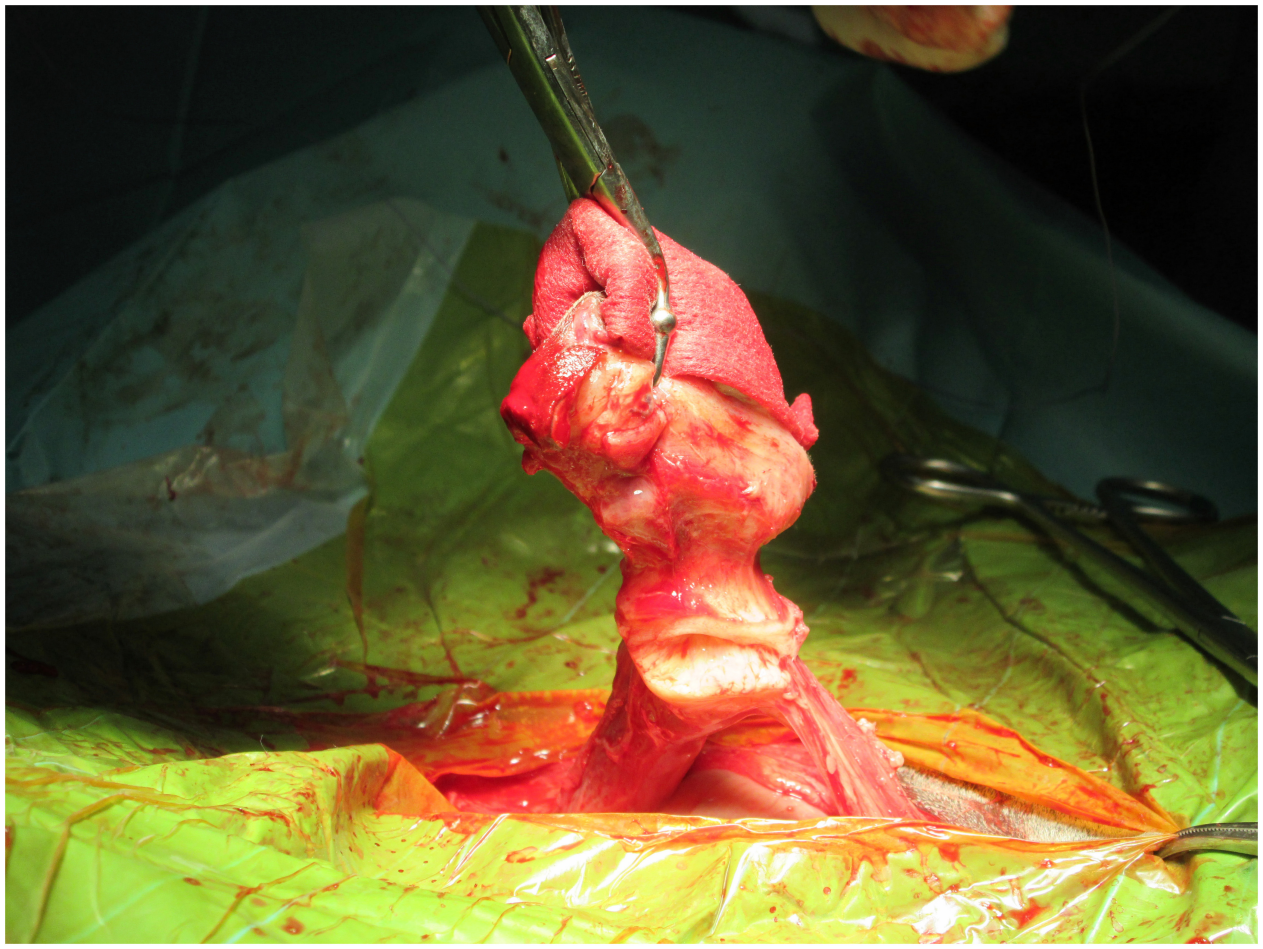
En bloc resection of urachus and umbilical remnants in a case of patent urachus.

Surgery is particularly warranted to remove the source of infection when there is an inadequate response to antibiotics like in cases of septicemia or joint infection (9). Also, when an abscess is detected by ultrasound evaluation, surgery is recommended, as the penetration of antimicrobials into the abscesses is really challenging. The surgical approach may differ based on the infection's severity (19). Surgical treatment alternatives consist of various methods, such as omphalectomy via en-bloc resection, in which external and internal umbilical remnants are removed (Figure 4), or laparoscopically assisted resection of the umbilical vein and arteries (5, 8).

### 2.3 Surgical treatment

Nowadays, the most common technique used for surgical resection of infected umbilical remnants is the en-block resection previously described (20, 21) as follows: Surgeons double ligate the vein cranial to all abnormal tissue. Then a transection and dissection of the vein free of the surrounding fascia using Metzenbaum scissors and blunt dissection is done. It is necessary to identify the umbilical arteries tracking caudally on either side of the urachus. Retraction of the umbilicus (together with the now ligated and transected vein) caudally will allow visualization of the arteries and urachus and facilitate retraction of the bladder. Then a placement of stay sutures just dorsal to the apex of the bladder and the level of the planned resection is performed. It is necessary to double-ligate and transect the arteries and the apex of the bladder to ensure the complete removal of the urachus together with the rest of the umbilical remnants, which can now be removed from the surgical site. This technique has shown good survival rates, with no complications described during or due to the procedure itself. However, in more recent cases, bipolar electrocautery device (BED) (Ligasure Atlas^®^) has been used to transect the umbilical arteries and umbilical vein after placement of transfixion ligature (5).

When performing omphalitis surgery, it is important to evaluate which of the umbilical structures are involved. In the research conducted by Codina et al., which included surgical procedures on 82 foals, the urachus emerged as the most commonly affected structure, with a prevalence of 84.1% (5). Additionally, 51.2% of the foals exhibited infection in at least one umbilical artery, 39% showed infection in the umbilical vein, and 59.8% presented with multiple affected structures. These findings align with those from Oreff et al. (8), who similarly noted that urachal enlargement was the predominant surgical finding at 71.3%. However, in contrast to Codina et al., Oreff et al. reported a greater frequency of enlargement in the right umbilical artery (52.3%) compared to the left umbilical artery, which had an incidence of 35.4%. Furthermore, they observed a higher percentage of foals with multiple affected structures, recorded at 68.6%. Conversely, Rampacci et al. (11) found that the umbilical vein was the most frequently affected structure among the foals subjected to surgery, occurring in over 50% of the 31 foals operated on. In conclusion, there is variability in the most commonly affected structure within previously reported research, but all agree that there is frequently more than one affected structure.

In case that en-bloc resection does not facilitate the complete removal of infected tissues, as if significant infection of the umbilical vein or hepatic abscesses exist, marsupialization of the umbilical vein has been proposed as an option (15). Two distinct techniques have been detailed based on the type of stoma created: cranial midline or right paramedian translocation (5, 14, 15, 22). Long term survival described in 11 cases was impressive (91%) and the primary long-term complication observed, occurring in 3 out of 10 cases, was the formation of hernias at the marsupialization site. However, no correlation was found between the marsupialization technique employed and the occurrence of hernias. When compared to simple en-bloc resection, marsupialization may necessitate longer hospitalization, additional veterinary care, and consequently, higher associated costs (15).

Laparoscopically assisted resection of the umbilical structures was first described in 1999 by Fischer (23). In this study, 11 foals underwent laparoscopically assisted resection while under general anesthesia in a dorsal recumbent position. No significant complications were noted. Minor complications observed included slippage of the endoscopic ligation clip and bladder laceration, both of which were successfully addressed during the procedure. The advantages of this technique include reduced postoperative morbidity, quicker recovery, smaller incisions, and enhanced intraoperative access to the structures.

Short-term outcomes in foals with patent urachus have been described as promising, with survival rates between 77% and 91% (5, 7, 8, 23). Nevertheless, in a study that considers outcomes after umbilical resection (5), including not only patients with patent urachus but also with umbilical remnant infection or with both conditions, it is described that failure of passive transfer and longer anesthesia times were associated with increased post-operative complications. Pre-existing septic arthritis and/or physitis and other post-operative complications were also associated with decreased survival. Therefore, these factors should be considered when recommending surgery in foals with patent urachus or infected umbilicus. One of the main limitations of this study was that it did not specifically differentiate outcomes or complications among foals within each of the groups. However, in the group of foals treated with only patent urachus, there were fewer non-survivors, which may be indicative of a better surgical prognosis vs. foals with only umbilical remnant infections or those with patent urachus and umbilical remnant infections.

Several reports have addressed the prognosis in foals with omphalophlebitis, signaling the number and type of complications related to it as the main factors involved (7, 18). Previous reports following different surgical techniques, such as en-block resection, laparoscopy, and/or marsupialization of the umbilical vein, describe survival rates to hospital discharge of 77%, 91%, and 100%, respectively (5, 8, 14, 15, 22).

Concerning short survival (to discharge from the hospital), Codina et al. described that 89% of foals were discharged alive (5), consistent with findings from earlier research (7, 8, 11) long-term survival rates following umbilical remnant transection, a study on short and long-term outcomes after umbilical vein marsupialization (15) noted that 10 out of 11 foals discharged from the hospital were alive at a median follow-up time of 44.5 months after the surgery.

Regarding complications, septic arthritis emerged as the main condition associated with reduced survival rates (5, 8, 11). Additionally, Codina et al. noted that the occurrence of postoperative complications was associated with decreased survival rates, with the development of new septic arthritis and/or physitis following surgery being the only postoperative complication that correlated with survival outcomes (5). Oreff et al. pathologies, elevated creatinine levels, increased heart rate, extended hospitalization, and a longer duration from arrival to surgery (8).

After laparoscopically assisted resection of the umbilical structures was first described (9, 23) there has been no publication providing further information about the prognosis of foals treated laparoscopically, nor neither an article comparing foals treated by en-block resection vs. laparoscopically assisted resection, in terms of surgical management, complications during a surgical procedure or derived from surgery and survival rates. Although there is a lack of information regarding the aforementioned, this technique has been demonstrated to be highly valuable as no major complications were described, and minor complications could successfully be dealt with during the procedure. Furthermore, laparoscopy has important benefits that must be taken into account when planning a surgery, as it allows smaller incisions and increased access and visualization of intraabdominal structures. As it allows better visualization and access to intraabdominal structures, this technique could be interesting in cases in which extensive umbilical vein infection is suspected. The use of laparoscopically assisted resection of extensive umbilical vein infections could lead to lower hernia formation rates described in cases of marsupialization of the umbilical vein (8, 14, 15). However, using this technique requires additional skills not used routinely and hands-on training by the surgeon, limiting its use to those who can use it safely and skillfully. Furthermore, it should be noted that general anesthesia is required for the procedure.

## 3 Uroperitoneum

Uroperitoneum most frequently arises following bladder rupture during parturition in colts, though cases have also been noted in fillies. The incidence rate varies between 0.5% and 2.5% among foals admitted to equine hospitals (5, 24–27). Ruptures may occur in the urachus or on either the dorsal or ventral side of the bladder and tears on the ventral surface commonly extend longitudinally toward the bladder neck (25, 28, 29). Typically, bladder tears measured on the dorsal surface range from 2 to 5 cm and feature hemorrhagic and edematous margins (24), although it has been described defects with smooth edges and no signs of traumatic disruption (24, 27, 30, 31). Foals with neonatal maladjustment syndrome can be predisposed to have bladder ruptures, as these foals may have alterations in detrusor muscle tone that complicate complete bladder emptying. Any pressure on the abdomen of these foals may lead to bladder rupture; therefore, in foals with any suspicion of inadequate detrusor function, placement of an indwelling catheter for a few days is recommended (5). Urachal rupture, on the other hand, is frequently a consequence of urachal infection (28, 29).

Initial indicators of uroperitoneum consist of a reduction in nursing vigor and lethargy, which are followed by progressive abdominal distension and intermittent signs of colic (25, 26). Foals often exhibit hyponatremia, hypochloremia, hyperkalemia, metabolic acidosis, and azotemia, and may also experience concurrent sepsis (32, 33). When there is a urachal defect near or outside the body wall, the buildup of urine within the abdominal fascia and subcutaneous tissues results in significant swelling and localized edema around the umbilicus (5).

**Figure 5:**
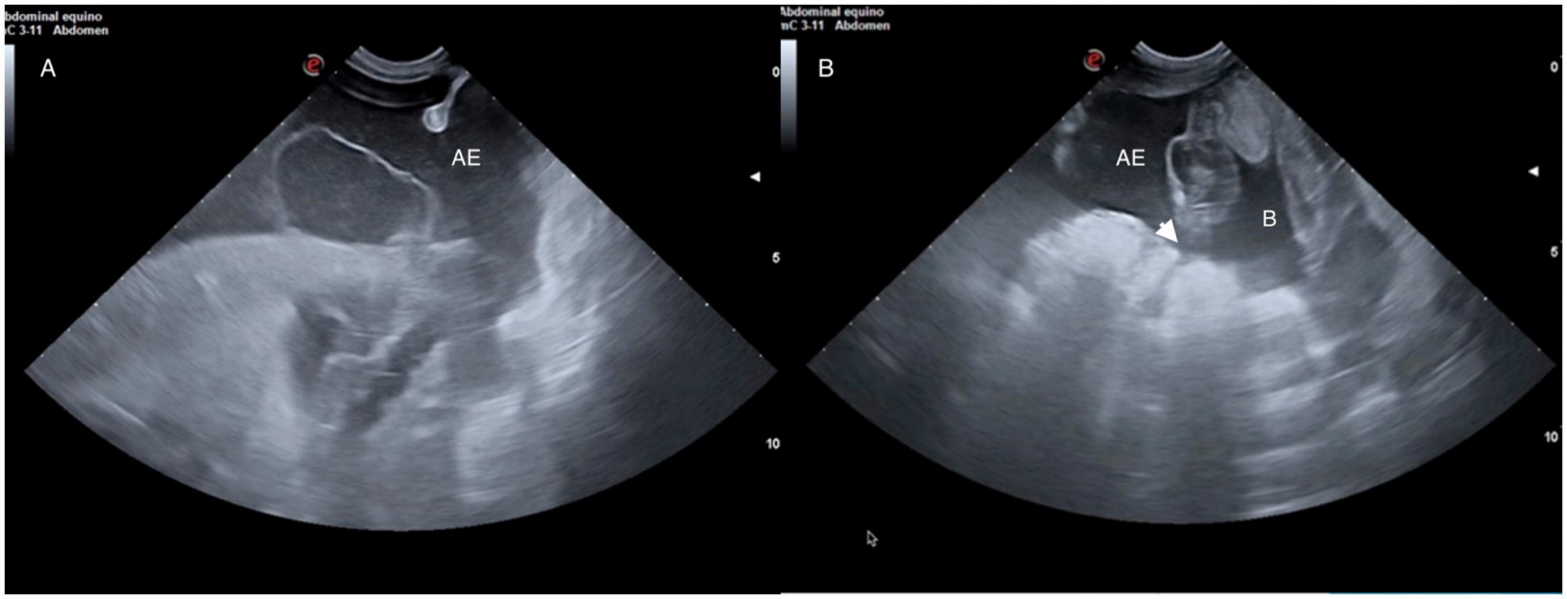
**(A)** Ultrasound image of an abdominal effusion (AE) due to uroperitoneum. **(B)** Ultrasonographic image of a bladder **(B)** with thickened walls and a discontinuation (white **arrow head**) on them compatible with a bladder wall defect.

Transabdominal ultrasonography has proven to be the most important imaging modality in the diagnosis of the disease (32, 34) (Figure 5). An increase in anechoic-free abdominal fluid would raise suspicion of uroperitoneum. In earlier case series (32), bladder wall defects were visible in 10 out of 25 cases (40%). In a more recent publication (35), a discontinuity in the bladder wall was observed in only 18 % of the foals. The ultrasonographic finding that led to the conclusion of a bladder wall defect was the discontinuity in the smooth and echoic bladder wall. However, it is difficult to assess a tear in the bladder sue to its volumetric anatomical variations. Therefore, the absence of a bladder wall defect on ultrasound should not exclude surgery, as in most cases it is not visible. The initial approach to assess that free abdominal fluid would be to obtain a sample by an abdominocentesis in the ventral abdomen. This procedure has both purposes: firstly, it enables the collection of a sample for subsequent analysis, and secondly, it helps to reduce the volume of free abdominal fluid.

A comparison of creatinine concentrations in serum and peritoneal fluid is the gold-standard for the diagnosis of uroperitoneum. While urine urea quickly diffuses through membranes and into the abdominal cavity, creatinine, being a larger molecules, remains largely confined to the peritoneal space. Therefore, peritoneal fluid to serum creatinine ratios >2 are indicative of urine presence in the peritoneal cavity (36).

Uroperitoneum is a medical, but not surgical emergency. The two primary issues that frequently require urgent intervention are hyperkalemia and abdominal distension with urine. Significant hyperkalemia (exceeding 5.5 mEq/L) may manifest through subtle muscle tremors or cardiac arrhythmias (25, 37). Addressing hyperkalemia involves mitigating the effects of potassium on excitable membranes, shifting potassium from extracellular to intracellular compartments, and facilitating its elimination from the body (38). The objective of medical stabilization is to reduce serum potassium levels to below 5.5 mEq/L before proceeding with surgical intervention (25).

**Figure 6:**
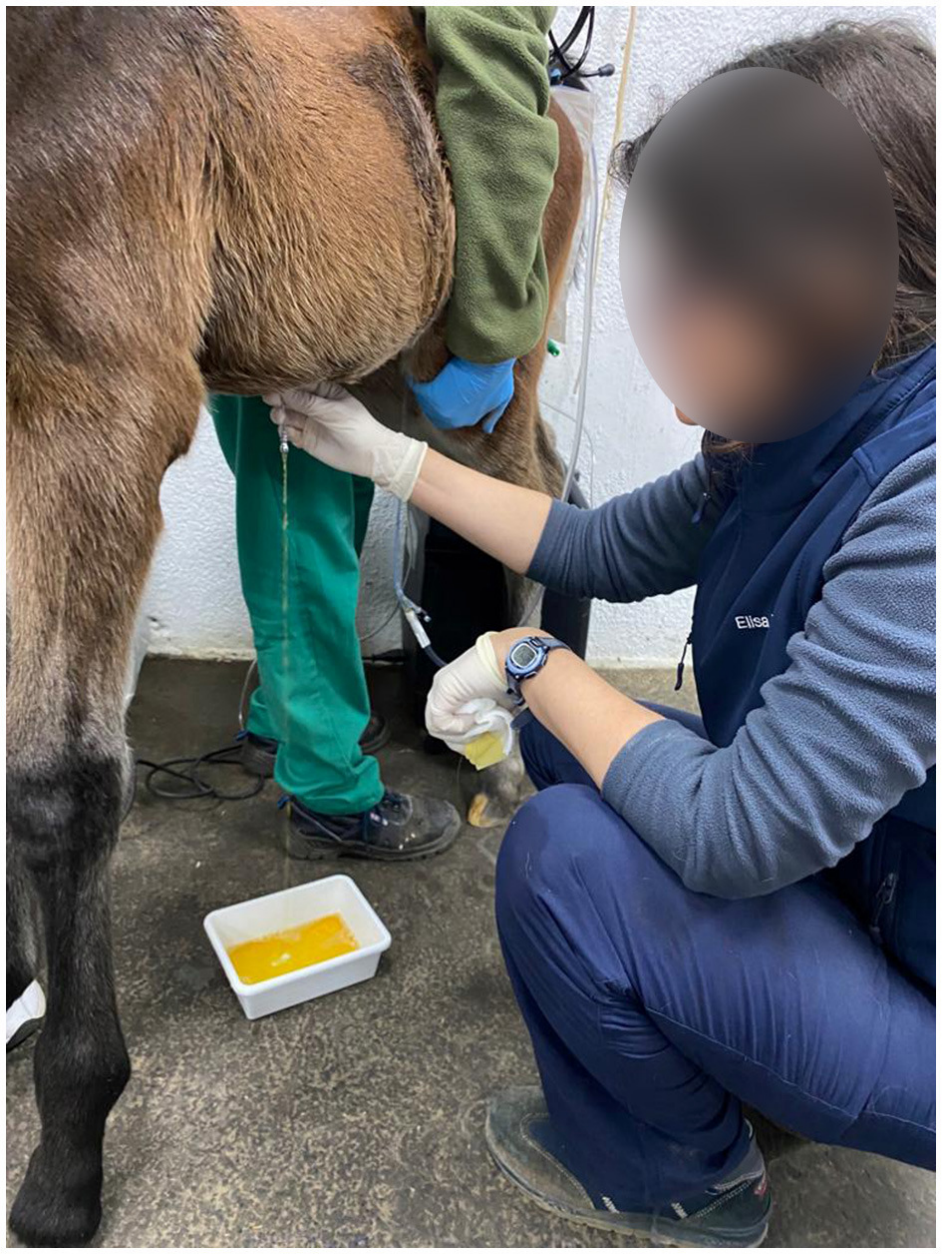
Peritoneal drainage of uroabdomen through a teat cannula in a case of uroperitoneum in a foal.

Drainage of urine from the abdomen is also essential to remove a large amount of potassium from the body as well as to decrease pressure on the diaphragm to allow adequate ventilation (Figure 6). Foals that have accumulated a large volume of urine in the abdomen may also develop pleural effusion that can complicate ventilation during surgery (39). Although accumulation of urine in the abdomen has been incriminated in the development of chemical peritonitis (30), most non-septic foals and adult horses that undergo successful surgical repair of the bladder have had favorable long-term outcomes without developing abdominal adhesions (39).

Cystography through the ventral midline incision provides direct access to the source of the uroperitoneum, thereby facilitating surgical techniques to seal off the source of urine leakage. The bladder can be distended with retrograde instillation of a sterile solution of fluorescein or methylene blue through the urethral catheter (29). Gas distention can also be of help to visualize the leakage from the bladder in the abdomen.

**Figure 7:**
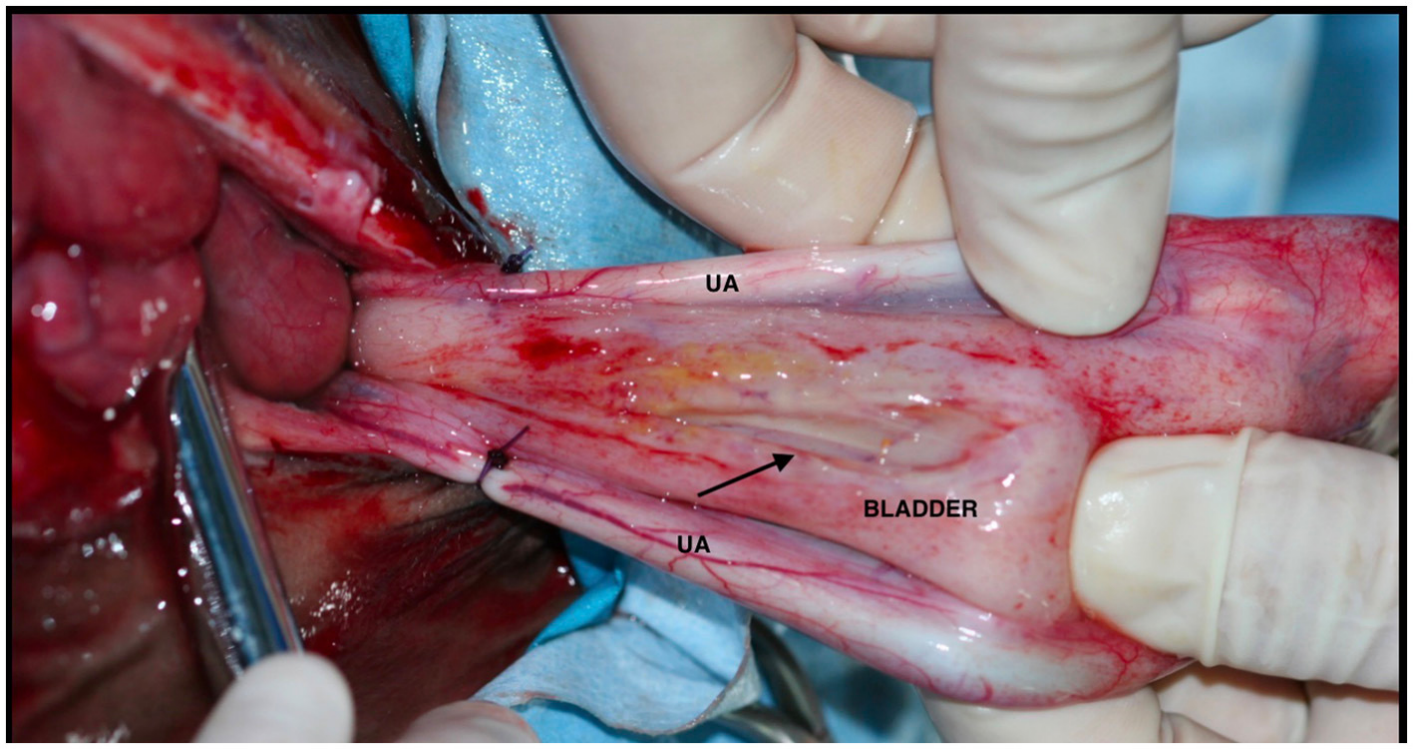
Bladder rupture (**arrow**) and ligation of the umbilical arteries (UA).

After identifying the tear (Figure 7), various authors have suggested removing the wound margins (29) before cystography. However, excising the margins may not always be feasible, as the location of the tear can make resection impractical. For instance, tears that are hard to access or those that affect a considerable length of the bladder may not be appropriate for margin debridement.

The use of double-layer inverting pattern closure or an interrupted pattern in the first layer, avoiding the vesicular mucosa, followed by a continuous inverting pattern is widely accepted for surgical closure (40). Previous studies reported that double double-layer closure in ovine cystotomies had a higher leaking pressure than single single-layer closure, independently of the suture material used (41). Other reports have encountered uroperitoneum recurrence using both double-layer closures (42). Including the mucosa layer in the holding layer through the submucosa in the bladder carries the risk of contact with the alkaline urine of the bladder, which could result in premature hydrolysis of the suture or calculus formation (16, 43). The cases that have developed calculus formation have been related to multifilament sutures in a double inverting pattern, non-absorbable sutures, and staples (16, 42, 43). In comparison, the authors observed a reduced incidence of calculus formation when employing absorbable monofilament sutures in a double inverting pattern closure. It is recommended that a urinary catheter be used within the first 72 h following surgery (42) to facilitate the healing of the bladder defect.

Alongside the previously mentioned treatment options, laparoscopic closure of the bladder in foals has also been documented (44). In one case, a bladder rupture was detected during a laparoscopic examination of the abdomen in a foal that developed uroperitoneum. The defect was located at the apex of the bladder, positioned between the attachment of the urachal remnant and the remnant of the left umbilical artery. This lesion was subsequently repaired with a laparoscopic stapling device, allowing for minimal intervention and excellent visualization (44). Given the benefits of laparoscopy as a therapeutic approach, which include advantages inherent to the procedure itself, it could be advantageous to visualize the caudal region of the abdomen and conduct a tension-free dissection (45). The use of barbed suture for laparoscopic bladder repair has been evaluated *ex vivo* (46, 47), although it has not been evaluated in foals nor has it been available long enough to know if it contributes to calculi formation. Up to date, only one report utilizing a stapling device (44) resulted in the development of calculi.

While surgical repair is frequently effective, complications after surgical repair do occur (46, 47). Two cases of uroperitoneum recurrence 72 h after surgery have recently been described (48). Surgical correction of these lesions was not performed due to the location of the primary lesions (a tear in the dorsocranial margin of the bladder and a tear in the pelvic urethra). Both cases were treated medically with abdominal drains and urinary catheters, and the foals were discharged and survived the short-term follow-up. However, one of the foals was readmitted for investigation of colic signs 2 months postoperatively and was euthanized due to the presence of multiple adhesions between the small intestine and the abdominal wall.

The acute complications of uroperitoneum include the development of severe ventricular arrhythmias during anesthesia (1, 25). It is anticipated that some degree of chemical peritonitis will occur because of urine exposure. However, septic peritonitis is an uncommon occurrence (25, 32, 33). The observed recurrence rates of uroperitoneum after primary repair vary between 12% and 27% (27, 42). Clinical signs, such as free abdominal fluid, abdominal distension, or drainage from the abdominal incision, have been noted 48 h post-initial repair. The predominant complications stem from suture dehiscence or inadequate closure of the defect (27, 42). The literature reports a wide range of survival rates after hospital discharge, from 56% to 86% (16, 25 29, 32, 33, 42). It is possible that these figures have been affected by the presence of additional illnesses, such as positive sepsis scores, which have been linked to reduced survival rates of 57% (32). In that study, sepsis was identified as a significant risk factor for recurrence, occurring in 80% of cases. As recent research (42) indicates, the recurrence of the condition is not solely dependent on whether the foal had a positive or negative sepsis score.

These survival rates, when followed over longer periods (e.g., 6 months or more), indicate slightly lower long-term survival rates (69%) (25, 49). Despite encouraging short-term survival rates, the risks of complications and recurrence, in foals with concurrent sepsis must be carefully managed to ensure better long-term outcomes.

## 4 Ureteral tears

In cases where the underlying cause of the uroperitoneum is not evident due to the presence of intact bladder and urachus, a ureteral tear should be considered as a potential diagnosis (38, 50–55). It represents an uncommon cause of uroperitoneum that may manifest bilaterally and has been reported in male and female foals of various breeds (25, 52–54). These animals tended to be several days older (4–16 days of age) than those with urachal or urinary bladder lesions. The pathophysiology of ureteral defects in the foal remains poorly understood, with histopathologic evaluation suggestive of acquired defects (28, 38, 50–55).

In cases where a ureteral defect is found in the retroperitoneal space, a significant amount of anechoic fluid can be observed in the kidney area through ultrasonographic examination. However, the diagnosis is more frequently established by ruling out other causes of uroperitoneum, as previously mentioned. Additional diagnostic techniques may be needed to define the site of the tear and to identify if single or multiple defects are present (56). The use of cystoscopy with catheterization of the ureters and injection of a dye, such as methylene blue during exploratory laparotomy, may allow for demonstration of a ureteral rent. In some cases, computed tomography (CT) urogram, which clarifies the excretory phase and the collecting system of the urinary system, has facilitated the detection and diagnosis of ureteral lesions (38). Primary repair of these defects using small (5–0) suture with and without a stent has been reported but in cases of ureteral tears where the lesion is situated in the dorsal region within the retroperitoneal space, nephrectomy has been employed as a treatment option (38).

Overall, this review emphasizes the importance of monitoring and appropriately managing foals at risk for uroperitoneum, especially during and after parturition. Further research is needed to better understand the mechanisms and improve clinical strategies for diagnosis and treatment. The literature presents a notable variation in survival rates following hospital discharge, ranging from 56% to 86%. These statistics suggest that various factors, including the presence of comorbid conditions, significantly influence outcomes. While immediate survival rates may be optimistic, careful management of complications and recurrence in those foals with concurrent sepsis is essential to improve long-term survival outcomes. This underscores the need for comprehensive care strategies during the post-discharge period.

## 5 Ectopic ureter

Ectopic ureters represent the most prevalent congenital anomaly within the equine urinary tract (UT). These can occur unilaterally or bilaterally; however, both cases are uncommon in horses (57). Fillies seem to have a higher susceptibility to this condition, while in foals, it may often go undiagnosed due to the challenges in recognizing urinary incontinence in males. Although a specific breed predisposition has not been established, some researchers believe that Quarter Horses may be at an increased risk (58).

The primary underlying cause is the abnormal embryologic development of the metanephric bud. Ectopic ureter formation can occur if the ureteric bud (metanephric bud) does not integrate into the urogenital sinus, fails to migrate upward toward the bladder neck, or if there is a failure in the regression of the mesonephric duct. In the first case, the ectopic ureter may open near the urethral papilla in females or into the pelvic urethra in males. The second scenario, which is exclusive to females, results in the ureter potentially opening along the vagina, cervix, or uterus (58).

Clinical signs linked to this condition include urinary incontinence and its consequences, such as urine scalding of the perineum and hind limbs, pollakiuria, and/or dribbling. However, these signs can be more challenging to identify in colts, as urine entering the pelvic urethra may sometimes flow retrograde into the bladder. While most foals, particularly fillies, demonstrate some level of incontinence from birth, they generally remain healthy and perform well (59). In rare instances, abnormal renal function may arise, especially if the condition is bilateral or accompanied by other congenital urinary anomalies (60).

Diagnosing unilateral cases can be more difficult, as foals might show normal urine flow; however, these situations are typically easier to manage. The definitive method for diagnosis has been the direct observation of the ectopic ureteral opening through endoscopy. Initially, a cystoscopy is conducted to locate one or both ureteral openings, monitoring for intermittent urine flow from them. If an opening cannot be located, the endoscope should be withdrawn to check for ectopic openings in the urethra, vestibule, or vagina. In some instances, the use of intravenous dyes such as sodium fluorescein, indigotindisulfonate, azosulfamide, or phenolsulfonphthalein may assist in identifying the ectopic ostia by coloring the urine to aid identification. (58). In fillies, the vagina or vestibule is explored (the vagina is inflated with air and the vulva is sealed to improve visibility) while for males, this entails examining the urethra (61).

Additional diagnostic methods include transabdominal ultrasonography of both the ureters and kidneys (62), as it is common in fillies to observe ureteral distention alongside the presence of an ectopic ureter (63). Furthermore, other techniques such as anterograde or retrograde ureterography, along with CT or MRI (57, 62, 64–66) have also been reported, in addition to nuclear scintigraphy for assessing renal function prior to surgery (65, 66) However, before any surgical intervention, it is crucial to ascertain whether the ectopia is unilateral or bilateral and its exact location, as well as to confirm normal urinary function and the absence of urinary infection (67).

The treatment for ectopic ureters in horses is exclusively surgical, with various procedures available tailored to the individual case. A comprehensive assessment of the UT is also important to identify any other developmental anomalies (68).

Addressing any existing urinary infections and evaluating the functionality of both kidneys are crucial steps as well. In cases of bilateral ectopic ureters, it is necessary to assess urethral sphincter competency and bladder volume before proceeding with surgery (57). Cystometrography has been utilized preoperatively in several foals to assess detrusor function (69, 70).

Historically, ectopic ureters have been treated using unilateral nephrectomy (71–74) or ureteroneocystostomy (65, 70, 75), which repositions the ectopic ureter into the normal anatomical location in the dorsal bladder wall. However, this latter procedure can be challenging if the ectopic ureter is abnormally dilated (>3 cm) or distorted, complicating surgical manipulation (59, 61, 62, 70, 76, 77).

In cases where only one side is affected, ureteronephrectomy is recommended upon the diagnosis of renal conditions such as ipsilateral pyelonephritis, unilateral hydronephrosis, or congenital renal dysplasia (75), which are frequently observed in fillies over 4 weeks old (2). Various techniques for nephrectomy are described, including the ventral midline or flank approach with rib resection, conventional laparoscopic surgery, and more recently, hand-assisted laparoscopic nephrectomy (62, 78). Laparoscopy presents several benefits, such as a decreased likelihood of postoperative complications associated with ventral midline incisions, reduced risk of peritonitis, minimal disturbance to the abdominal cavity, lower anesthesia-related risks, and a quicker recovery time. However, in younger foals, as previously mentioned, laparoscopies are also performed under general anesthesia.

Concerning the uretero-vesical anastomosis in horses, three techniques have been outlined: a side-to-side anastomosis with ligation of the ureter's distal portion (77), an extravesicular end-to-side anastomosis connecting the severed ureter to a small cystotomy (69), and an intravesicular anastomosis that utilizes a large ventral cystotomy alongside submucosal tunneling of the ureter (69, 77).

A recent review comparing nephrectomy vs. ureterocystostomy (39), analyzed 14 cases treated with ureterocystostomy, resulting in successful outcomes for 10 cases, while 4 horses faced postoperative complications. Additionally, a case involving an initial ureterocystostomy in a Shire filly was documented (62), but the stoma's failure necessitated a hand-assisted laparoscopic nephrectomy to address the incontinence. In contrast, 4 horses with unilateral ectopic ureters that underwent unilateral nephrectomy were reported to have survived, indicating potentially more favorable outcomes with nephrectomy in similar situations (39). Surgical complications reported include acute peritonitis and adhesion formation. However, recent advancements in surgical techniques and equipment development are expected to enhance the success rates in correcting ectopic ureters.

Minimally invasive techniques in equine surgery are constantly advancing, and a thorough understanding of the anatomy in these areas is crucial for achieving successful results. A recent study on the retroperitoneal perirenal space (79) offers valuable insights to enhance the techniques required for addressing ectopic ureters and related issues. An extensive preoperative assessment and thoughtful selection of the correct surgical approach are vital for optimizing outcomes in the treatment of ectopic ureters in equine patients, thereby ensuring renal function preservation and enhancing the quality of life for affected horses.

A minimally invasive technique for ureteral ostioplasty has also been performed on two fillies with unilateral ectopic ureters (80). This procedure entailed the inflation of the urethra and bladder with air, followed by the application of a laparoscopic scissor or vessel sealing device to make an incision at the ectopic ureteral ostium in the urethra. The incision was made longitudinally from the ureteral ostium along the intramural ureter, extending to the normal entry point of the bladder. Both fillies experienced positive postoperative outcomes at 20 and 9 months post-surgery (80). This technique was feasible in these situations because cystoscopic examination revealed that the distended ureters were passing through the bladder wall, which confirmed their intramural position, as opposed to an extramural one. The difference between intramural and extramural ureters is crucial, as it influences the potential success of ureteral ostioplasty.

The most important issue to contemplate before deciding which technique to use in the face of an ectopic ureter is whether the problem is unilateral or bilateral and the position of the ectopic ureter about the bladder. Considering this, and of course, addressing an unaltered urinary function, several surgical options have been described. However, the main problem that surgeons face in these cases is that most of the information related to those procedures is scarce and old. That complicates enormously the choice of the procedure, as many of the complications described could probably be overcome nowadays by surgical techniques and equipment advances in the field. Also, the use of minimally invasive techniques like laparoscopy in selected cases has shown a promising alternative to traditional techniques. Therefore, more recent studies would be needed to compare outcomes and possible complications among different techniques. Meanwhile, the characteristics of the ectopic ureter and the surgeon's personal choice and skills will probably determine the selection of the procedure to resolve this uncommon pathology.

## 6 Hydroureter

Congenital and acquired UT abnormalities, such as hydronephrosis (dilation of the renal pelvis) and hydroureters (dilation of one or both ureters), are well-recognized conditions in humans but are rarely reported in animals (81). Unilateral or bilateral hydroureters, frequently associated to hydronephrosis have been described in foals (63, 65, 82). They can cause severe hyponatremia and subsequent severe neurological signs (83) and possibly colic pain (84).

The cause of these problems in horses is not completely known, although in some cases, ureteral obstruction is blamed for it (84, 85), but neurogenic and/or developmental defects could also be involved in foals (86). In human neonates, the causes of this syndrome include structural and non-structural abnormalities, such as pelviureteric junction obstruction, vesicoureteric junction obstruction, ureteral motility disorders and vesicoureteral reflux, among other UT abnormalities (87, 88).

Transabdominal ultrasound has proven useful as a screening procedure in diagnosing hydronephrosis and hydroureters in foals (84). However, fetal or postnatal ultrasound is not routinely performed in foals, making the occurrence and frequency of such abnormalities unknown. In a case, described by Nogradi et al. (85) hematuria was one of the clinical signs found and CT was recommended to further characterize these lesions and evaluate possible treatment options. Also, fluoroscopy has been used in the diagnosis of hydroureters, helping to perform a nephropyelocentesis and antegrade urography to detect the presence of hydroureters, ureteral stenosis and urethral defects. This technique is more routinely used in dogs, but it has also been described in foals (52).

Catheterizing the affected ureters and careful sodium correction would cause an improvement in laboratory abnormalities and clinical signs. However, these abnormalities may return once the catheters are removed. There may be less severe cases that recover with time and without catheterization (55). Nevertheless, long-term success is not usually favorable unless a physical obstruction affecting urine flow is identified and can be corrected (86).

In many occasions, hydroureters are associated with other urinary malformations such as ectopic ureters or other UT malformations (70). However, the use of CT is considered vital to determine the viability of a surgical intervention. Moreover, this technique allows us to assess the severity and likely etiology of the nephropathy as well as provide a more accurate prognosis to the owner before committing to the expense of surgery.

Definitive treatment may vary depending on the presence and type of other urinary disorders associated. Ureteroneocystostomy, as explained in the ectopic ureter section, has also been described as a treatment for this syndrome (52).

Considering the low prevalence of the disease, further studies are needed to determine the main causes of hydronephrosis and hydroureter. Based on recent publications, it can be concluded that these conditions are often secondary to other pathologies, primarily of umbilical origin, such as abscesses (81), but functional impairment of urine flow may also have contributed to these abnormalities. Cases of aortic aneurysm leading to secondary ureteral obstruction have also been reported. The prognosis in these cases is guarded, so a complete evaluation is always recommended, including imaging techniques, primarily ultrasound and, if possible, a CT scan, to characterize the lesions and assess potential treatment options.

## 7 Inguinal hernias

**Figure 8:**
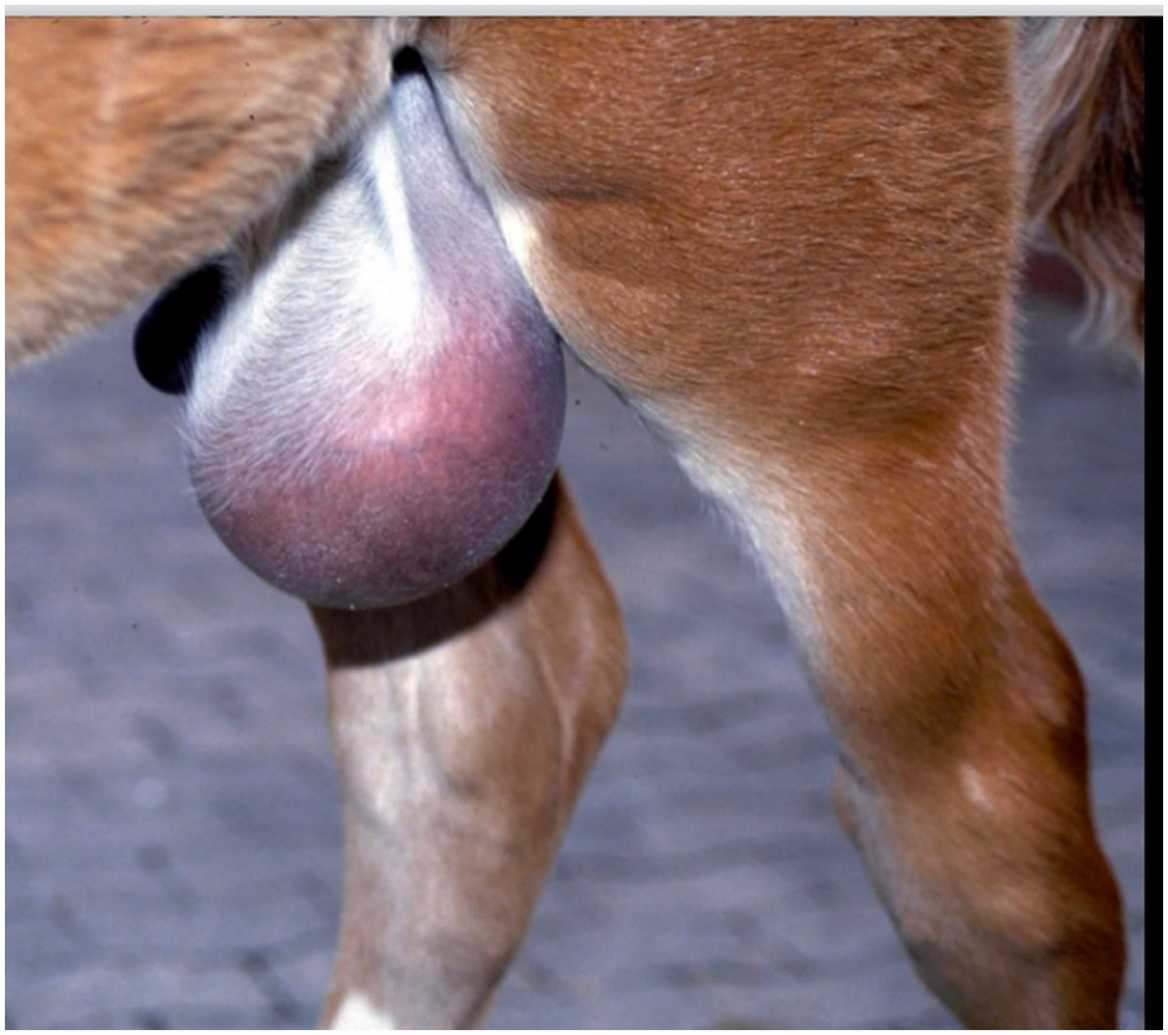
Inguinal hernia in a 30 days-old foal.

Inguinal hernias are identified by the extension of abdominal contents through vaginal ring. The herniated tissue is commonly a segment of the ileum or distal jejunum (89), although occurrences of inguinal herniation involving the large colon (90, 91) and urinary bladder (92) have also been reported. Herniation can be categorized as congenital or acquired. Congenital inguinal hernias are likely hereditary and may manifest unilaterally or bilaterally, with scrotal or inguinal swelling typically being the only visible clinical sign (Figure 8). In contrast, acquired hernias are usually found on one side. Diagnosis is generally established through physical examination and ultrasonography.

The most frequently observed condition in affected foals is a congenital indirect hernia, characterized by the passage of content through the vaginal ring into the vaginal process (93). This condition may arise from excessive growth of the extra-abdominal segment of the gubernaculum, leading to a vaginal process that has an unusually wide neck (94). It is important to differentiate this from the less common occurrence of a tear in the muscle wall or vaginal process, also known as a direct inguinal hernia, where the vaginal process tears and herniated intestines move into the subcutaneous tissue (89, 93). Surgical options for repairing a direct or ruptured inguinal hernia include open reduction or closed reduction under laparoscopic guidance with or without closure of the defect, provided that the intestine is viable. If the herniated intestine is non-viable, an open approach to facilitate resection and anastomosis if pursued by the owner is necessary (95).

**Figure 9:**
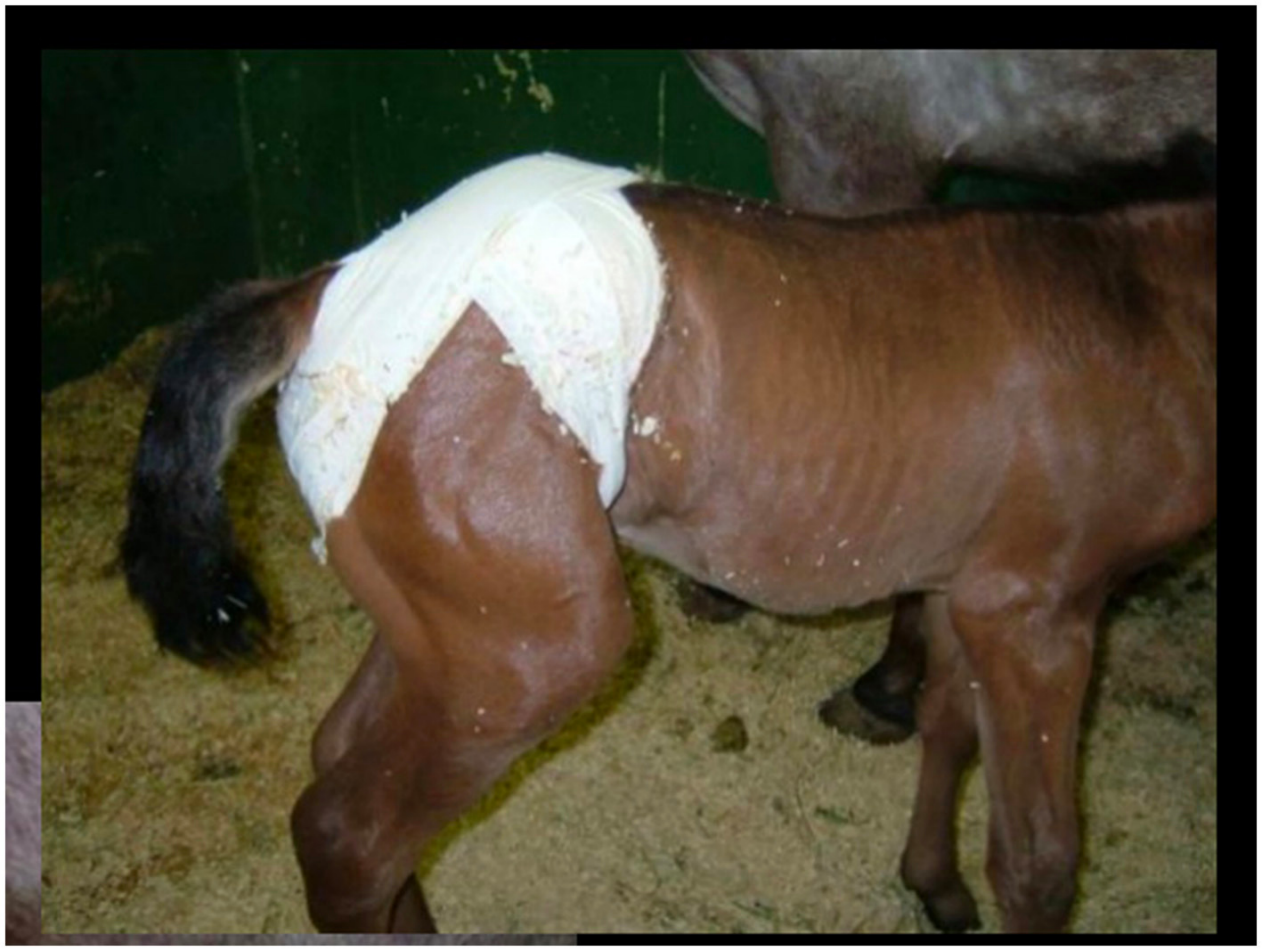
Application of a truss after manual reduction of an inguinal hernia.

Indirect congenital inguinal hernias are typically reducible and rarely lead to strangulating obstruction due to the large diameter of the inguinal rings, thus not resulting in abdominal discomfort (90, 93). These hernias often resolve with conservative treatment, involving manual reduction of the herniated intestine back through the ring until it closes spontaneously by 3–6 months of age. In certain cases, applying a truss after manual reduction may aid in recovery (Figure 9) (90, 93).

If conservative treatment proves ineffective, particularly when the hernia is non-reducible or exceptionally large (90, 93), or there is a ruptured inguinal hernia with signs of depression and abdominal discomfort (90, 96, 97), surgical intervention becomes necessary. This can be performed via an open inguinal approach (16) or through laparoscopy (3, 90, 98–104).

Treatment generally involves the reduction of the hernia, with or without unilateral or bilateral castration, and suturing of the external inguinal ring to prevent recurrence (91, 105). A direct inguinal approach is used for closed castration and closure of the superficial inguinal ring. If the hernia cannot be reduced, the herniated bowel is exposed for evaluation through a direct inguinal approach, and a ventral midline celiotomy may also be required. The non-strangulated bowel is returned to the abdomen, the testicle is removed, and the external inguinal ring is closed. In some cases, bowel resection may be needed when there is bowel involvement. The main potential complications include swelling, drainage, and recurrence of the hernia.

Laparoscopic techniques aimed at reducing the size of the internal inguinal and vaginal ring structures have become a favored treatment in recent years due to their numerous benefits, such as faster recovery, decreased pain, and lower complication rates compared to traditional open surgery. In laparoscopic procedures, the hernia content is retracted into the abdomen, and the vaginal ring is closed using intracorporeal suturing (98, 99), cylindrical polypropylene mesh prostheses (90), peritoneal flap hernioplasty techniques (3, 101), cyanoacrylate (100), a tacked intraperitoneal slitted mesh technique (104), a surgical anchoring system (103), or knotless barbed sutures (102).

Mariën et al. (106) presented one of the earliest laparoscopic techniques for closing the inguinal ring. Their study encompassed 12 cases of indirect and reducible hernias, along with 4 cases of ruptured hernia, involving foals aged between 1 day and 4 months. The procedure was performed under general anesthesia, where the herniated intestines were retracted into the abdomen using atraumatic laparoscopic forceps, and the peritoneal edges of the vaginal ring were secured with titanium staples. The dimensions of the staples after closure ranged from 5.3 to 3.7 mm, with 2–8 staples proving adequate to bring the free edges of the vaginal ring together. Drainage of the scrotal sac was only required in one instance involving a ruptured inguinal hernia, where an excessive accumulation of peritoneal fluid in the enlarged scrotal sac necessitated drainage to promote spontaneous regression. The postoperative recovery was characterized as painless and progressed smoothly. Follow-up evaluations conducted via telephone at 2 and 6–11 months after surgery indicated no issues in any of the cases.

In 2008, Caron et al. (98) described a technique for intracorporeal suturing to close the internal inguinal ring in six foals. The procedure was conducted under general anesthesia with the foals in dorsal recumbency. For foals with intact vaginal tunics, herniated viscera were reduced using Trendelenburg positioning, while in those with ruptured vaginal tunics, the herniated small intestine was retrieved by grasping and applying traction with atraumatic endoscopic forceps. Following castration, simple interrupted intracorporeal sutures made from synthetic absorbable material were employed to secure the closure of the internal inguinal and vaginal rings. Except for one foal, all the animals made a full recovery, with no reported complications or recurrence of clinical signs. Clinical follow-up was conducted over a period ranging from 6 to 17 months. One foal ultimately had to be euthanized due to a musculoskeletal issue, which was determined during necropsy to be unrelated to the surgical procedure.

Laparoscopic herniorrhaphy utilizing an automated laparoscopic suturing device in conjunction with barbed sutures has proven to be a safe and effective method for surgically addressing congenital inguinal hernias in colts. This technique was applied to nine foals with unilateral or bilateral hernias. At the owner's request, the testes of five foals were preserved. No intraoperative or postoperative complications related to the surgery were reported. The follow-up period lasted 8 months, during which no cases of reherniation were noted (76). Additionally, Vázquez et al. (102) recently published a retrospective study detailing 40 laparoscopic procedures in which inguinal rings were closed using barbed sutures alone or in combination with other techniques. Among these cases were three foals with congenital hernias, one of which presented a strangulated hernia accompanied by signs of colic pain. The foals were anesthetized and positioned in dorsal recumbency for the procedure. In those without colic, the hernias reduced spontaneously due to the effects of the Trendelenburg position and capnoperitoneum. It was sometimes necessary to have an operator externally support the testicles in the scrotal sac or to create an inguinal incision to grasp the testicle with forceps. All animals showed no complications during surgery or in the follow-up, yielding results comparable to those reported by Maurer et al.

The effectiveness of laparoscopic peritoneal hernioplasty using a peritoneal flap, cylindrical polypropylene mesh prosthesis, and intraperitoneal mesh with slits to anatomically close the vaginal ring and prevent future hernias has only been investigated in adult animals. This laparoscopic peritoneal flap hernioplasty technique was introduced in 2007 by Rossignol (101) in a study involving 13 animals, followed by Wilderjans in 2012 (3), who documented 30 cases treated with the same method. The procedure was performed standing under sedation. The peritoneum ventrolateral to the vaginal ring was elevated and incised on three sides, then detached from the underlying muscle, inverted, and secured dorso-medially and laterally to the parietal wall using intra-corporeal stitches or laparoscopic staples. The laparoscopic follow-up confirmed that the vaginal ring was effectively and completely covered in all cases except for one. No adhesions were noted, and none of the horses experienced a recurrence of inguinal hernia up to 4 years after the procedure (101).

Mariën (90) described the use of laparoscopic herniorrhaphy with cylindrical polypropylene mesh prosthesis in nine adult horses. A cylindrical polypropylene mesh was placed and secured within the inguinal canal. Within 2 weeks, this led to the formation of adhesions, resulting in an obliterated inguinal canal. A laparoscopic re-examination of three horses after 2 weeks showed obliterated inguinal canals but no adhesions. Additionally, five horses were observed to have multiple scar tissue lesions on the peritoneal surface around the internal inguinal ring.

The laparoscopic ring closure technique is a minimally invasive technique with few side effects according to published articles, so it seems to be the method of choice over conventional open surgery. Techniques to correct the hernia that preserve the testicle and prevent recurrence have been described, but laparoscopic methods, partially closing the inguinal ring, are probably superior. Consideration should be given to laparoscopic closure of the contralateral vaginal ring as well as the affected side if the horse has a future as a breeding stallion, because both sides could be at risk of herniation later (95).

While laparoscopic management of inguinal herniation of mostly adult horses has been reported, an open approach remains the primary approach for many equine surgeons for neonates and foals with inguinal herniation. The few studies that have reported laparoscopic repair in foals have not reported long-term outcomes nor has a comparison to an open approach been made. Though, short-term survival has been reported and remains good with laparoscopic treatment (98). It has to be considered though, that open surgical approach to treat those pathologies, especially in small foals could be more beneficial for them, following the personal experience of the surgeons writing this review, particularly if there is a compromise of the small intestine involved or resection is needed. Besides, laparoscopic technique in foals, unlike in adults, must be performed under general anesthesia, which can pose an added risk (98).

## 8 Conclusions

Making surgical decisions for foals with urogenital issues requires a meticulous evaluation of the severity of each condition, since these emergencies are typically life-threatening. A comprehensive patient assessment is crucial, as the prognosis can differ based on accompanying conditions or complications that may arise during hospitalization or after surgery.

Timely and precise assessment, along with a personalized approach, is vital for tailoring surgical interventions according to the severity of each patient's condition. Research indicates that outcomes improve significantly with proper preoperative management of metabolic imbalances and effective antibiotic therapy, which helps reduce complications and enhances survival rates.

In foals with a patent urachus or omphalitis, timely surgical intervention minimizes the risk of sepsis and alleviates short-term complications that may arise from ongoing leakage or failure of the urachus to close. Foals suffering from omphalitis need immediate and comprehensive evaluation of the affected structures, as infection has the potential to spread to adjacent tissues, liver, and joints. While mild cases may respond to medical treatment, surgery provides complete visualization and removal of infected tissues, lowering the chances of recurrent infections and improving the prognosis in cases of extensive infection.

Uroperitoneum is a critical condition that necessitates careful stabilization of electrolyte imbalances and other metabolic issues prior to surgery, as this can enhance survival rates by minimizing complications during anesthesia, surgery, and the recovery period. Small reducible inguinal hernias can be managed conservatively, particularly if the foal is stable. On the other hand, large or non-reducible hernias present a risk of strangulation, warranting surgical intervention. In such instances, prompt surgery is essential to prevent bowel ischemia and necrosis, and timely surgical management generally leads to a favorable prognosis.

Ectopic ureter and hydroureter are relatively rare urogenital conditions in foals. Evaluating renal function is crucial in both situations, and early surgical planning is necessary, particularly when there is a possibility to preserve renal function. Advanced imaging plays a vital role in these cases by clarifying the anatomy and functional status of the ureters and kidneys, facilitating decision-making for surgery. Surgical correction significantly enhances outcomes, although the prognosis can vary based on the extent of renal dysfunction prior to the operation.

Choosing between laparotomy and laparoscopy for the surgical management of urogenital disorders in foals necessitates a careful evaluation of the severity of the condition and a solid understanding of anatomical accessibility. While laparoscopy offers advantages associated with minimally invasive techniques, laparotomy remains the preferred approach for more complex or urgent cases, providing comprehensive access for the repair of affected tissues.
